# Grant Report on SCH: Personalized Depression Treatment Supported by Mobile Sensor Analytics

**DOI:** 10.20900/jpbs.20200010

**Published:** 2020-04-29

**Authors:** Jayesh Kamath, Jinbo Bi, Alexander Russell, Bing Wang

**Affiliations:** 1 Psychiatry Department, University of Connecticut Health Center, Farmington, CT 06030, USA; 2 Computer Science & Engineering Department, University of Connecticut, Storrs, CT 06269, USA

**Keywords:** depression, personalized depression treatment, mobile sensing, data analytics, machine learning

## Abstract

We report on the newly started project “SCH: Personalized Depression Treatment Supported by Mobile Sensor Analytics”. The current best practice guidelines for treating depression call for close monitoring of patients, and periodically adjusting treatment as needed. This project will advance personalized depression treatment by developing a system, DepWatch, that leverages mobile health technologies and machine learning tools. The objective of DepWatch is to assist clinicians with their decision making process in the management of depression. The project comprises two studies. Phase I collects sensory data and other data, e.g., clinical data, ecological momentary assessments (EMA), tolerability and safety data from 250 adult participants with unstable depression symptomatology initiating depression treatment. The data thus collected will be used to develop and validate assessment and prediction models, which will be incorporated into DepWatch system. In Phase II, three clinicians will use DepWatch to support their clinical decision making process. A total of 128 participants under treatment by the three participating clinicians will be recruited for the study. A number of new machine learning techniques will be developed.

## INTRODUCTION

Depression is a complex, heterogeneous, severely debilitating and chronic illness. It affects more than 264 million people worldwide, contributing to significant number of deaths by suicide every year [1]. The prevalence rate of depression varies across the world from 3% in Japan to 16.9% in the United States, with most countries reporting between 8% and 12% [2]. Due to its high lifetime prevalence and its effects on daily function and its mortality [3], depression is expected to become the world’s largest medical burden of disease by 2020 [4].

The goal in treating depression is achieving symptom remission and full functional recovery [5]. Similar to other fields of medicine, there has been a strong impetus in the field of psychiatry to personalize depression treatment, i.e., quickly identify the best treatment for a depressed individual while minimizing side effects in the clinical setting. However, despite decades of research, finding the perfect treatment for a patient has been elusive—very few clinical characteristics, biomarkers, or genetic variations have been identified that can reliably predict differential effectiveness or adverse effects of specific depression treatments [6–8]. Clinical guidelines such as the Canadian Network for Mood and Anxiety Treatments (CANMAT) Clinical Guidelines provide recommendations for treatments for major depression [9]. However, the guidelines acknowledge significant limitations of evidence-base, lack of comparative trials, heterogeneity of patient populations, and several other factors such as drug-drug interactions, pharmacodynamics and pharmacokinetic factors [9]. In the context of this limited evidence-base, guidelines recommend tailoring treatments to the patient, i.e., accounting for diverse factors in clinical setting and adjusting treatments as needed [10,11]. This “trial and error” treatment strategy can have serious detrimental consequences in clinical setting, e.g., negative impact on patient-provider therapeutic alliance, patients dropping out of treatment [12]. It has been reported that only about 35% of patients will remit upon initial treatment in a given episode [13]. On the positive side, for the patients who did not remit after the first treatment, up to 40% experience significant improvement after switching to an alternative treatment, adding a second medication, or adding psychotherapy provided they pursue these options [13]. The reality, however, is that patients typically drop out of treatment if initial treatments fail or they experience side effects: After starting antidepressant treatment, nearly half make no follow-up visits and only one quarter return often enough to pursue additional treatment options [12].

To optimize patient care outcome, it is highly desirable to predict whether a patient will eventually respond to treatment early in the course of the treatment. Recently, a particularly exciting discovery is that early improvement or lack thereof in the first two weeks after initiation of an antidepressant treatment is a good predictor of later full response to treatment [14–21]. This finding highlights the value of early assessment and close follow-up with patients, particularly important for those who have shown no improvement. In existing studies, close monitoring has been achieved using physician-administered follow-ups or patient self-administered questionnaires. While feasible in well-executed and well-funded studies, this is difficult to carry out in clinical settings for a number of reasons: Firstly, frequent follow-ups by clinicians is difficult due to the significant lack of trained professionals—in the United States, the ratio is 14.5 psychiatrists per 100,000; in developing countries, the ratio is more than ten times lower [22]. Secondly, patient self-administered questionnaires are burdensome, and responses to these questionnaires are often subjective and limited to recall bias. Commonly used depression questionnaires in the clinical setting such as the Patient Health Questionnaire (PHQ-9) ask patients to report their emotional status and other symptoms over preceding two week period [23]. Patient’s responses to these questions are frequently colored by their emotional status at the moment when they complete these questionnaires. Thus, responses to these questionnaires fail to capture objective data including day-to-day variations in patient’s depression status, its behavioral manifestations, and its impact on patient functioning [24]. Impairment in functioning is a critical depression criterion according to the Diagnostic and Statistical Manual of Mental Disorders (DSM V) and an important determinant of depression severity [25]. Depression questionnaires also fail to reliably and objectively capture change in depression severity, especially changes that occur in response to treatment initiation [24].

Smartphones, with their multiple sensors and increasingly advanced computing capabilities, have the potential to serve as “human sensors” by monitoring users in real time for changes in their behavioral patterns. Several reports provide evidence of feasibility and potential efficacy of using smartphone based data for clinical inferences in the management of affective disorders, primarily depression and bipolar disorder (by our team and other research groups, see reviews in [26–32]). Specifically, in the LifeRhythm Project, a 4-year project funded by the National Science Foundation, our group conducted a two-phase study in college age participants with depression (in comparison with a control group). Our results demonstrated that sensory data collected from mobile and wearable devices—without any user interaction—can provide critical information that correlates with depression symptoms, and can be used to automatically detect depression [33–38]. Specifically, in Phase I of the project, we developed a smartphone application, called LifeRhythm app, to passively collect sensory data (location, activity, social interaction) for both Andriod and iPhones, the two predominant smartphone platforms. We further developed feature extraction techniques to extract behavioral features from the sensory data as correlates of depression symptomatology, and machine-learning models to predict self-report questionnaire scores and depression status (i.e., whether one is depressed or not). These techniques and prediction models were then validated and refined in Phase II of the study. In addition, in Phase II, we further explored using wristbands (Fitbit devices), in addition to smartphones, for characterizing behavioral features that are correlated with depression.

The current project builds on the insights and experiences we gained from the LifeRhythm project. The goal is to develop a system, DepWatch, that leverages mobile health technologies as well as machine learning tools to closely monitor patients’ depression symptoms and assist clinicians’ decision making. Specifically, the system aims to provide timely assessment of depression symptoms through mobile data analytics. Such timely assessment of depression can help clinicians personalize treatment by (i) identifying patients who are failing treatments early, and (ii) assisting them to take necessary actions before patients drop out of treatment. The project comprises two studies. The focus of Phase I study is to extend the LifeRhythm app to include self-report questionnaires on mood, anxiety, medication adherence, and medication tolerability and safety. The self-reports and the passively collected sensory data will then be used to develop, evaluate and cross validate machine learning models for assessing depressive symptoms and predicting patients’ response to treatment in the future e.g., in the two, four or six weeks after treatment initiation. At the end of Phase I, we will develop a web portal that will assist clinicians’ decision making (e.g., to continue the current treatment or to change the treatment) by incorporating the machine learning models. The web portal will be developed in collaboration with clinicians, by closely seeking their input and feedback through focus groups. In Phase II study, the web portal will be used by clinicians to evaluate its usability and efficacy. We hypothesize that DepWatch system will be able to predict response/non-response to depression treatments by capturing change in behavioral patterns as it relates to changes in patient’s depression severity, which will be useful in clinicians’ decision making process.

This is a four-year project, currently in its first year. It is a joint interdisciplinary project between the University of Connecticut (UConn) and University of Connecticut Health Center (UCHC). It is led by Dr. Bing Wang, Dr. Jinbo Bi and Dr. Alexander Russell from UConn and Dr. Jayesh Kamath from UCHC. The project is broadly related to the studies that use smartphones and wearable devices to monitor, manage and assist the treatment of affective disorders (see reviews in [26–32]). As an example, MONARCA I and II trials feature patient self-monitoring using both objective sensory data and subjective self-assessment on smartphones; in addition, the data can be visualized on a web portal that can be accessed by both patients and clinicians [39,40]. The studies in [41,42] explore context-sensitive intervention delivery via smartphones to people with depressive symptoms to provide them in-situ support. Our study focuses on providing clinicians a decision support system that helps them to evaluate and adjust depression treatment, leveraging mobile sensor analytics.

### Aims of the Grant

#### Aim 1

Develop DepWatch system. DepWatch collects sensory data passively from smartphones and wristbands, without any user interaction, and uses simple user-friendly interfaces to collect ecological momentary assessments, medication adherence and safety related data from patients. The collected data will be fed into machine learning models to be developed in the project to provide weekly assessment of patient symptom levels and predict the the likelihood of whether a patient will respond in the next several weeks. The assessment and prediction results are presented using a graphic interface to clinicians to help clinicians make treatment decisions.

#### Aim 2

Extract higher-level structures from sensing data. While low-level features directly extracted from sensing data (e.g., entropy of locations, the amount of time spent at home) are correlated with depression, the correlation tends to be low. This project will identify features that provide semantic information or higher-level structures on user behaviors, which are potentially more correlated with depression symptoms. Specifically, the features to be explored include environment context (e.g., restaurant, movie theater, work place, gym) and routines that a user follows regularly that can describe the structures of the user’s life.

#### Aim 3

Develop new machine learning algorithms. The focus will be on (i) longitudinal prediction, which is necessary since response to a medication may only be assessable after a period of time and the depression symptom change of a patient at the current time can depend on the features in the past, and (ii) multi-task feature learning for the challenging setting with large-scale heterogeneous data.

## BACKGROUND AND HIGH-LEVEL APPROACH

### Background

The current practice for treating depression is a “trial and error” approach that commences a course of medication and requests that patients to return to evaluate the effects later on. In the absence of emergency situations i.e., a patient calls due to severe side effects or suicidal ideation, the interval between two visits is typically 4 weeks. While the guidelines may recommend shorter intervals, more frequent follow-ups are difficult in clinical setting due to shortage of resources, particularly limited availability of psychiatrists. The above practice has several drawbacks. First, the clinician has no way of monitoring symptom evolution of the patient until the next follow-up visit. Even at that time, the symptom reports are based on questionnaires, which are subjective and known to suffer from recall bias [24]. The delayed awareness of a patient’s status may lead to delayed change in treatment strategies, potentially leaving a patient to more impairments associated with depression, higher risk of suicide, and higher likelihood of discontinuing treatment prematurely. In addition, this practice does not leverage the evidence that the presence or absence of early improvement (within two weeks after starting a new treatment strategy) can predict the treatment outcome (full response or not) in the future.

There is an urgent need of a system that (i) provides objective measurements and assessments to clinicians frequently e.g., on a weekly basis, with minimal burden to patients; and (ii) predicts treatment outcome (i.e., whether continuing the current treatment is likely to lead to remission later on) based directly on brief subjective report and objective behavior data so as to help clinicians best make treatment decisions.

### High-Level Approach

We will develop DepWatch, an automatic data collection and analytics system, to support and inform clinicians’ decision making for depression treatment. Development of machine learning models for the DepWatch system uses both QIDS-SR (Quick Inventory of Depressive Symptomatology-self report) scores [43] and clinician assessment as ground truth. However, a higher emphasis is placed on the clinician assessment which includes review of weekly QIDS scores and participant interview on a monthly basis or twice per month if clinically indicated. The QIDS has been validated in clinical settings. It provides more details on depression symptoms than another widely used self-report questionnaire, PHQ-9 [23]. Specifically, QIDS separates certain symptoms into individual items, e.g., insomnia into initial, middle, and terminal insomnia; appetite into low vs high appetite; weight into low vs high weight; and psychomotor symptoms into agitation vs retardation. PHQ-9 combines each of these sets of symptoms into one item. For our study purpose, i.e., to develop prediction models on the various depressive symptoms, it is critical to separate these symptoms. At the same time, QIDS is still not too burdensome and reflects the DSM V diagnostic criteria [43].

**Figure 1.**
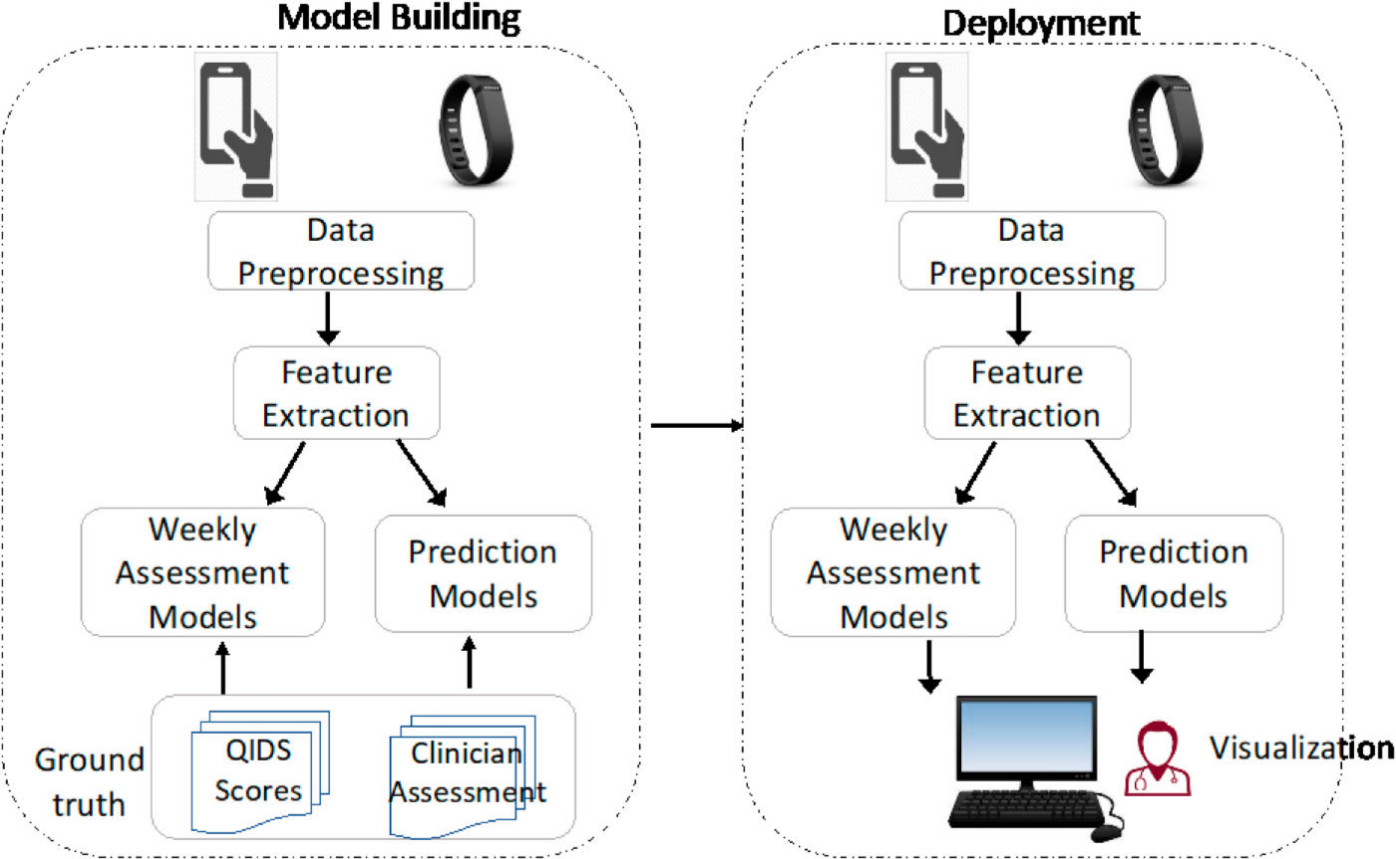
DepWatch: high-level approach. The ground truth includes self-reported QIDS (Quick Inventory of Depressive Symptomatology) scores, and Monthly clinician assessment (including review of weekly QIDS scores and participant interview).

Figure 1 illustrates how DepWatch works: It collects patient data, encrypts the data, and sends it to a secure server. At the server side, the data will be preprocessed and analyzed with machine learning algorithms (that have been previously trained) to directly assess the patient’s current status. The results can be visualized by the clinicians using a graphic interface. More importantly, the results are primarily based on objective data gathered by DepWatch, capturing changes in behavioral patterns as it relates to changes in patient’s depression status. A clinician then logs into DepWatch regularly to review the status of his/her patients e.g., on a weekly or biweekly basis, and can leverage these analytics to help decide whether the current treatment plan needs to be changed. In this case, the clinician will ask the patient to come back for a more detailed evaluation or connect with patients by phone to discuss ongoing treatments. In addition, the clinicians can receive automated alerts if their patients’ exhibit behavior that suggests a significant negative change in symptoms or questionnaire responses that indicate suicidal ideation or cessation of antidepressant use, e.g. due to side effects or due to lack of efficacy.

Through DepWatch, a clinician can monitor a patient on a continuous basis, and change treatment plans in a timely manner to avoid adverse impacts on patients due to delayed adjustment of treatment plans. Since only the patients that need to be evaluated earlier, as identified by DepWatch, will be called back (the rest come back at their regularly scheduled visits), the clinic resources are used more efficiently. When a patient comes back for a follow-up visit, the clinician already has detailed information on the patient’s mental health status to make better decisions. As a result, the treatment for patients is more personalized. From a patient’s point of view, DepWatch allows a patient to be more engaged in the treatment process. As a result, we hope that patients will be more willing to continue their treatment and seek alternate medications if necessary.

## STUDY DESIGN AND DATA COLLECTION

The study is organized around two phases (see the timeline of the project in Table 1). The two primary goals of Phase I are (i) data collection and (ii) development, training, and cross-validation of the machine learning models, which will be incorporated in DepWatch system. In Phase II, we will arrange for clinicians to actively use the DepWatch system developed in Phase I to evaluate usability and efficacy. Subject recruitment of both phases will proceed through IRB-approved announcements and procedures. In this section, we briefly describe the study procedures; more details on the technical challenges are deferred to later sections.

Phase I study will recruit a total of 250 participants from several UConn Health outpatient clinics and from surrounding communities. Participants will meet the following inclusion/exclusion criteria: age 18 and above, have unstable depression of at least moderate severity as defined by a score of ≥11 on the QIDS questionnaire [24], initiating a pharmacological treatment (monotherapy or adjunctive treatment) for depression, no current or past diagnosis of bipolar disorder or primary psychotic disorders such as schizophrenia, and no clinically significant medical, psychiatric, or substance use comorbidities that may adversely affect participant’s study participation and/or affect their adherence to study protocol e.g., significant cognitive deficits, clinically significant substance use disorder within one month of study enrollment. The participation of each participant will last up to 12 weeks. All subjects meeting the study eligibility criteria will complete the QIDS at baseline. At the baseline visit, i.e., at the time of enrollment, participants’ demographic and clinical information will be collected e.g., medical and psychiatric comorbid conditions, past treatment history/medication trials. Participants will be asked to download and use the LifeRhythm app on their smartphones. The LifeRhythm app was developed in a prior project to passively collect objective sensory data, and is extended in this project to collect brief questionnaires, including QIDS (weekly), mood and anxiety (daily), medication adherence, medication tolerability and safety (weekly). Participants will also be provided a Fitbit wristband (if they do not have one) to collect specific physiological information, e.g., sleep, heart rate, activity. Monetary incentives/compensation will be provided to the participants for their participation and adherence to study procedures. The study clinician will follow up with a patient once per month (or twice per month if clinically indicated) to correlate QIDS reports with patient’s verbal reports through phone call or secure video call. The study clinician will closely monitor patients for worsening of depression symptoms, and will coordinate communication with the patients’ regular clinicians if significant worsening of symptoms with emergence of active suicidal thoughts with intent and plan noted based on study-related assessments.

In the Phase II study, three clinicians will use DepWatch to support their clinical decision making process. A total of 128 participants (aged 18 and above, each treated by one of the three clinicians) will be recruited for this study phase. The inclusion/exclusion criteria will be the same as for Phase I. For half of the participants, their clinicians will use DepWatch system, i.e., they will be prompted to review patient data/assessments gathered by the study team, and use the prediction model to assist their clinical decision when appropriate. The other half of the participants will serve as the control group; their clinicians will not use DepWatch system during their treatment. Changes in treatments initiated by the clinicians in response to the reports provided to them and overall depression outcomes for both groups will be assessed.

## MACHINE LEARNING MODELS AND DATA ANALYTICS

In Phase I study, we will use the collected data to train a family of new machine learning models. These machine learning are in two broad categories: (i) time-series models for predicting QIDS scores and clinical depression, e.g., remission, mild depression, severe depression, and (ii) direct prediction of changing depression status, e.g., significant improvement, mild improvement, no significant change, etc. The first category of models are trained directly against collected QIDS scores and clinical diagnosis, and aim to predict these via a variety of collected data e.g., sensory data, demographic information, past treatment history, mood/anxiety data. The second category of models are geared towards directly detecting significant changes in depression during the few weeks after initiation of treatment, one of the principal goals of this project. While changes in depression can be detected based on the prediction of QIDS or clinical diagnosis i.e., through the first category of models described above, we anticipate that higher reliability can be achieved by directly training our models against the associated “categorical” clinical ground truth. Specifically, we will perform two types of predictions: (i) determining whether a patient’s status is worsening, remains to be about the same, has improved, or has improved significantly, and similarly determining a patient’s status in specific symptoms e.g., interests, sleep, psychomotor; in both cases we also wish to identify the specific features relevant to the prediction, and (ii) predicting longer-term trajectory without changes in treatment, i.e., the likelihood of improvement in the next two to four weeks.

One significant challenge in developing the above machine learning models is that the data are complex and heterogeneous, involving diverse types of objective sensory data (e.g., location, activity, social interaction) collected on different platforms (e.g., Android, iPhones, Fitbit), subjective self-reports (e.g., mood, anxiety, medicine adherence), and clinical data (e.g., history of medication). In addition, the prediction targets are not directly compatible: QIDS score is a numerical outcome, while depression severity is a categorical outcome. We envision that multi-task feature learning (MTFL) methods are necessary and beneficial to our setting. Specifically, we can use MTFL methods to construct sparse mappings from the heterogeneous data to the multiple targets of predicting QIDS scores and depression severity categories. Our prior study [36] developed a MTFL method that jointly builds inference models for related tasks of different types, including classification and regression tasks, based on sensory data, which will be expanded to include even more heterogeneous data sources in this study.

Another significant challenge in our study is that the data is longitudinal, and the data records collected at different points in time are not independently and identically distributed. Specifically, because response to a medication may be visible or assessible only after a reasonable accumulation of time, the depression symptom change of a patient at the current time point could depend on not only the data records of the current time period e.g., the current week, but also on the data collected in the past e.g., the past week, two weeks, or even longer periods of time. Building predictive models using longitudinal records that are not independently and identically distributed is challenging. We plan to leverage statistical methods to correct for such samples. In an early study [44], we applied a variant of such methods to study the fMRI data from patients of Alzheimer’s Disease using a series of historical fMRI images that were taken at 24, 18, 12, 6, and 3 months in the past. We envision such an approach will be promising for this study.

The accuracy of the above machine learning models depends heavily on (i) data quality and sample size, and (ii) feature extraction. In our prior LifeRhythm study, we have found that missing sensory data is a prevalent and severe problem [35]. The problem will be further amplified in the current project due to the more diverse data that will be collected. The missing data problem becomes more challenging to address if certain subjects miss an entire view of data (e.g., a self-report, an entire day or multiple days of location data) rather than sporadic absence of a few measurements. Classic multiple imputations or matrix completion methods are clearly ineffective here because no information in the specific data source can be used to impute data for such samples. The commonly-used strategy of simply removing samples with a missing view or missing variables can dramatically reduce sample size, thus diminishing the statistical power of subsequent analyses [35]. We plan to leverage approaches based on deep learning models for data imputation. Specifically, we envision a promising direction is generative adversarial network (GAN) [45], which learns to generate new data with the same statistics as the training set. In addition to data imputation, we will further develop robust feature extraction techniques that provide features to the machine learning models. In earlier work [34–38], we have explored using various features from location and activity data e.g., entropy of locations, the amount of time spent at home, circadian movement, similarity in location across days. While these features are correlated with depression, the correlation tends to be relatively low. In this study, we will identify features that provide semantic information or higher-level structures, which may be more correlated with depression. One direction is to identify environment context, e.g., restaurant, movie theater, work place, shopping plaza, outdoors, gym, social places, which are important in correlating with depression symptoms. Another direction is identifying higher-level routines that a user follows regularly and can describe the structures of the user’s life.

## SYSTEM DEVELOPMENT AND EVALUATION

The DepWatch system contains two main components: (i) a data collection system that collects both objective sensory data and subjective patient self-reports to a secure server, and (ii) a web portal that visualizes patient information to clinicians to help their decision making. In the data collection system, the LifeRhythm app will be deployed on participants’ phones to passively collect diverse sensory data (location, activity, social interaction) and brief self-report questionnaires, such as the daily cognitive assessment and weekly assessment of medication adherence, tolerability and safety. The self-report questionnaires use easy-to-use graphic interface and participants are sent notification to fill in the questionnaires at the due date directly on his/her phone. The data collected by the app will be encrypted at the phone, and transmitted to the secure server when the phone is connected to a WiFi network. Each participant is assigned a random ID. The collected data is associated with the random ID, instead of the real identity of the user. The Fitbit data is collected (after user authentication during the informed consent) using the Fitbit Subscription API provided by Fitbit to the secure server.

The web portal will display three main categories of information. The first category is the information that has been gathered, including behavioral features (e.g., the amount of time spent at home each day, the amount of time in bed each day), mood (e.g., mood category each day), tolerability and safety in a specified time period (i.e., the past 2 weeks). The goal is to present a patient’s information to the clinician in an easily accessible form. The second category presents evolution of depression symptom (e.g., improving or worsening) in the general status and specific symptoms (e.g., interests, sleep, psychomotor) for a specified time period (e.g., in the past four weeks, relative to the baseline, or relative to the previous week). The third category is the prediction results, i.e., the likelihood of whether a patient will respond in four weeks with current treatment. Note that the results for the latter two categories are obtained from our machine learning models; they do not use QIDS reports at all (indeed QIDS reports are only collected for training and cross-validation the machine learning models; patients do not need to fill in QIDS when DepWatch is used in practice). The web portal needs to be designed so that it can be easily incorporated into clinical practice and be useful to clinicians’ decision making. We will design it in close collaboration with clinicians, seeking their input and feedback through focus groups, e.g., what are the most important information to display, how to display the information, etc. The design of the web portal will go through an iterative process, with multiple rounds of design and refinement. In Phase II study, three clinicians will use the web portal to view information about their patients who elect to participate in the study as well as the prediction results. We will encourage them to send us comments while using the system, and will improve the system based on their comments. Especially, we will seek their feedback and thought process when their clinical judgement differs from the results presented by the system. In the middle and at the end of Phase II study, we will ask the clinicians to evaluate the usability, accuracy, and efficacy of the system, which will be used for improving the system in the future.

Note that DepWatch system serves primarily as a decision support system for clinicians. It does not serve as a tool for patients self-monitoring. As a result, the design in the LifeRhythm app mainly focuses on providing easy-to-use interfaces for patients to enter the questionnaire information at the due date. It does not provide interfaces for patients to keep track of their past input. The web portal will only be used by the clinicians. Extending the system to provide information to patients is left as future work.

## CURRENT STATUS

**Figure 2.**
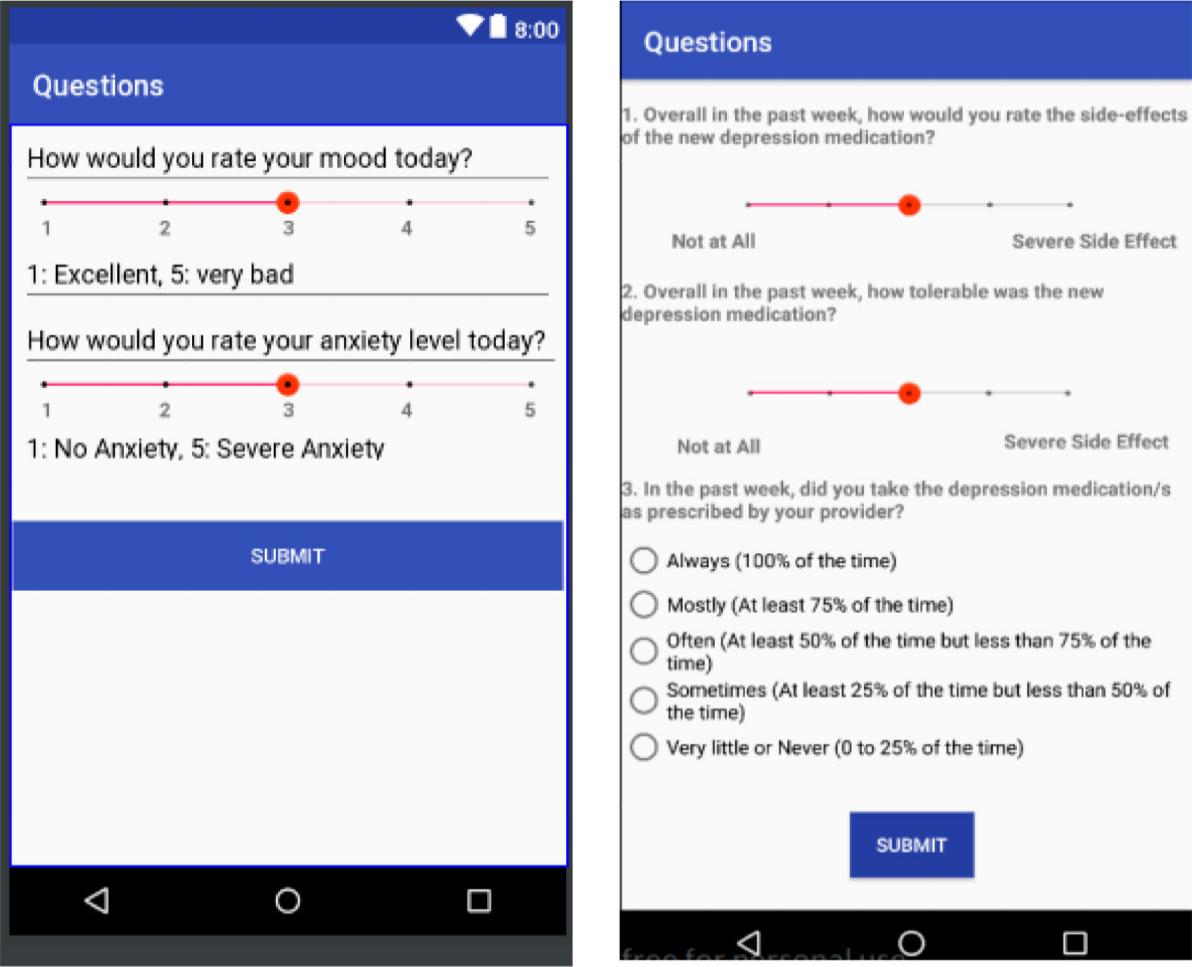
Two daily questionnaires on mood and anxiety (**left**) and three weekly questionnaires on safety, tolerability, medication adherence (**right**).

The project started in August 2019. We have completed developing the data collection system. Figure 2 shows the two daily questionnaires (on mood and anxiety) and three weekly questionnaires (on safety, tolerability, medication adherence) that have been developed in the LifeRhythm app. The app runs in the background, passively collecting sensory data. When it is the time to fill in the questionnaires, notifications will be sent to the users to remind them to fill them in. We stated recruiting participants for Phase I study in January 2020. As the data are being collected, we will analyze the data and develop, train and cross validate machine learning models to provide symptom assessment and predict the trajectory of response.

## CONCLUSIONS

Current treatment of depression lacks strong evidence-base, objective and timely assessments, and treatment biomarkers. DepWatch, a mHealth system developed by our team, captures behavioral patterns as a correlate of depression status. The ongoing project investigates its utility to predict response/non-response to depression treatment in real-time. DepWatch will provide clinicians truly objective data on their patients depression status. Furthermore, such data provided in real-time will serve as a behavioral biomarker helping clinicians make critical treatment decisions. Development of DepWatch is a vital step towards personalized and patient-centric depression care.

## Figures and Tables

**Table 1.** Timeline of the project.

Tasks	Year 1		Year 2		Year 3		Year 4	
	1–6 months	7–12 months	1–6 months	7–12 months	1–6 months	7–12 months	1–6 months	7–12 months
Develop data collection system								
Phase I study recruitment								
Develop and evaluate machine learning models								
Develop web portal								
Phase II study recruitment								
Summarize lessons learned								
